# Computer Simulation of Whole-Body Vibration in Port Container Handling Machine Operators

**DOI:** 10.3390/s25206346

**Published:** 2025-10-14

**Authors:** Ricardo Luís Alves Silva, Kleber Gonçalves Alves, José Ângelo Peixoto da Costa, Alvaro Antonio Villa Ochoa, Roberto Nobuyoshi Junior Yamada, Paula Suemy Arruda Michima, Gustavo de Novaes Pires Leite, Álvaro Augusto Soares Lima

**Affiliations:** 1 Department of Mechanical Engineering, Federal University of Pernambuco, Cidade Universitaria, 1235, Recife 50670-901, Brazil; ricardoalves@recife.ifpe.edu.br (R.L.A.S.); kleber.gbalves@ufpe.br (K.G.A.); roberto.yamada@ufpe.br (R.N.J.Y.); paula.michima@ufpe.br (P.S.A.M.); 2 Department of Higher Education Courses (DACS), Federal Institute of Education, Science and Technology of Pernambuco, Av. Prof Luiz Freire, 500, Recife 50740-545, Brazil; angelocosta@recife.ifpe.edu.br (J.Â.P.d.C.); gustavonovaes@recife.ifpe.edu.br (G.d.N.P.L.); 3 Department of Mechanical Engineering, Universidade Federal do Rio Grande do Norte, Campus Universitário-Lagoa Nova, Natal 59078-970, Brazil; alvaro.augusto@ufrn.br

**Keywords:** whole-body vibration, musculoskeletal disorders, modal and harmonic analysis, occupational hygiene, finite element method, WBV, FEM, ergonomics of static posture

## Abstract

This study aimed to evaluate the effect of whole-body vibrations (WBV) on ergonomics related to static posture during the operation of container handling machines (Portainer). A 3D numerical model of a seated man was developed using modal and harmonic analysis based on the finite element method (FEM), and implemented on the ANSYS platform to achieve this. Quantitative analyses of whole-body vibrations were carried out in actual workplaces at a port terminal in northeastern Brazil, considering the interaction between the human and the machine. A comparison was made between the real data collected at the operating sites and the values obtained from the developed model. Concerning vibration exposure, the results revealed a critical situation: in 86.2% of cases, the Acceleration of Resulting Normalized Exposure—A(8)—exceeded the alert level, and in 96.6% of cases, the Resulting Vibration Dose Value (VDV) also surpassed this threshold. Similarly, an alert level was exceeded in 97.0% of cases. According to the maximum limits established by Brazilian legislation, the acceleration from normalized exposure did not exceed the limit, while the resulting vibration dose value was surpassed in 20% of cases. The modal analysis results helped identify the critical directions of vibration response, thus supporting the assessment of human exposure effects and the structural performance of the system analyzed. The harmonic analysis showed good agreement between the model and the real acceleration data in the frequency range of 3 to 4 Hz.

## 1. Introduction

Data from the United Nations Conference on Trade and Development—UNCTAD [1] indicate that around 80% of global trade is transported by sea, which has led to an increase in cargo volumes in ports due to global trade expansion at a rapid pace. Inevitably, it has an impact on the work shifts of port machine operators [2,3].

Due to their peculiar operating characteristics, Portainer—or quay cranes—are essential in the loading and unloading of containers from ships. As a result, due to the high demand for cargo handling by sea, port operations for loading and unloading ships generally run in uninterrupted shifts, causing their operators to work for long periods without a break [4,5]. Long shifts expose operators to whole-body vibrations, increasing the risk of spinal musculoskeletal issues [6,7], which is why they are increasingly being recognized as an occupational risk to human comfort, performance, and health itself [8,9].

According to the Brazilian Ministry of Health (2019), exposure to occupational vibration is responsible for a significant portion of cases of Repetitive Strain Injury (RSI) and Work-Related Musculoskeletal Disorders (WMSD), directly implying damage to the quality of life and job performance of workers, hazard to body structures such as muscles, tendons, ligaments, cartilage, bones, joints, and nerves [10,11]. Musculoskeletal disorders are also an important health problem with major socioeconomic impacts on society [12,13]. WMSDs were responsible for $54 billion in costs and a third of all lost working days in the United States [14] while in Brazil, the Ministry of Health reported that RSI and WMSD combined accounted for 67,599 cases in the last 10 years, an increase of 184% over the period [15].

Numerous studies are therefore being carried out to understand, assess, and numerically model the various impacts associated with whole-body vibrations of users [16]. Charles et al. [17] recommend that key ergonomic factors be considered in any vibration assessment, as vibration is just one of many pathogens. Fatigue and musculoskeletal pain can affect postural control, which can increase the risk of errors and can result in reduced quality of work or production, as well as dangerous situations. In the same context, Lorenzino et al. [18] affirm that whole-body vibration is an important stress factor that can strongly affect the user’s experience of comfort in certain environments and can cause physical disturbances to the user.

Using field data, Lynas and Burgess-Limerick [19] conducted a study to obtain data on the types of equipment used in mines—such as shuttler cars, personnel transport vehicles, and load-haul-dump machines during regular operations, as well as the vibration amplitudes experienced by operators. Data was collected from three types of mining equipment in Australia. It was consistently observed that vibration levels exceeded the danger thresholds defined by the ISO standard, particularly in personnel transport vehicles. Good practices such as road maintenance, reducing vehicle speed, and replacing seats were shown to significantly reduce vibration exposure.

The ISO 2631 standard for Whole-Body Vibration (WBV) defines health risks using a Health Guidance Caution Zone (HGCZ) rather than strict danger thresholds. This zone is based on the weighted root mean square (RMS) acceleration A(8) over an 8 h period and ranges from 0.45 m/s^2^ (lower limit) to 0.9 m/s^2^ (upper limit). Exposures below 0.45 m/s^2^ are considered safe, exposures within the zone require caution, and exposures above 0.9 m/s^2^ are likely to present health risks [20].

In a related study on WBV in the agro-industrial sector, Adam et al. [21] studied the effects of sitting posture and vibration magnitude on the suspension system of an agricultural tractor seat. WBV magnitudes were measured in 11 individuals seated in different postures on the tractor seat system. The results revealed three peaks in seat transmissibility, with the primary resonance occurring between 1.75 and 2.5 Hz for all postures. In turn, Adam et al. [21] analyzed how the height of the tractor seat affects lateral vibrations on the operator. The results showed that a 30 cm reduction can decrease lateral vibrations by 20%, increasing comfort without affecting vertical vibrations. Predicting lumbar well-being is essential in occupational health, as it allows for the development and implementation of accurate ergonomic guidelines for operating industrial tractors, aiming to mitigate the effects of whole-body vibration. In this context, Sing et al. [22] conducted a comparative study to identify the most effective model for estimating the Static Compression Equivalent Dose Daily (S_ed_) in tractor drivers. The study assessed classical regression models, ensemble models, and meta-models. Results indicated that the SLR-E (Stacking with Linear Regression) ensemble model provided the best prediction of S_ed_, achieving an R^2^ of 0.93. Average operating speed was found to be the most influential parameter for this prediction. Similarly, Oncescu et al. [23] presented a simulation methodology to evaluate the dynamic vibrational behavior of the operator-vehicle system in an autonomous electric agricultural tractor, focusing on vibration modes and natural frequencies in accordance with ISO 2631-1. This approach integrated a validated nine-degree-of-freedom biomechanical model of the operator seated. A detailed modal analysis was performed in three stages (tractor, seat, seated virtual dummy) using the finite element method (Altair Sim Solid) to optimize vehicle performance and reduce operator exposure to vibrations. The system’s natural frequencies occasionally matched hazardous resonance zones (below 5 Hz). The tractor seat’s suspension natural frequency was 3285 Hz. Vibration mode 5 of the dummy (operator), at 4.03 Hz, demonstrated a strong interaction with vibrational excitations. Singh et al. [24] designed and tested an ergonomic seat for self-propelled agricultural machinery intended to reduce operators’ WBV exposure. The seat design integrated anthropometric data and piezoelectric vibration isolators. It was developed and tested under laboratory conditions for various seat types and engine speeds. To optimize the WBV reduction, an artificial neural network combined with genetic algorithms was employed to identify the minimum vibration values. The results indicated that the optimal engine speed was 1421 rpm for seats without isolators and 1460 rpm for those equipped with isolators.

Aiming at mobility comfort, Jiménez-Canos et al. [25] conducted an experimental analysis on a low-displacement motorcycle under different suspension load conditions, making it possible to observe the vibration response using a triaxial accelerometer. The analysis was carried out in the time, frequency, and time–frequency domains. The results showed that the frequency of interest remained below 20 Hz (the natural frequency range of the human body) in all cases studied. Tatsuno et al. [26] investigated how exposure to Whole Body Vibration in vehicles affects the disturbance of static standing balance function after driving, establishing a link with slip, trip, and fall accidents in the land transportation industry. The study proposes that the ISO 2631-1 standard [27] should incorporate loss of standing balance as a health indicator for assessing WBV exposure. Based on this observation, the study proposes that a rest period of approximately 4 min may be adequate for balance recovery after 60 min of exposure to 0.5 m/s.

Xu et al. [28] used the Finite Element Method (FEM) to study the response of spinal parameters in five adults with degenerative scoliosis exposed to cyclic vibration. They created a numerical finite element (FE) spine model and compared results between a scoliotic spine and a non-scoliotic one. The simulation revealed that the scoliotic spine is more sensitive to occupational vibrations, exhibiting more resonance frequencies. This increase in resonance can amplify the spine’s response to cyclic vibrations, potentially worsening spinal deformities.

Using the same numerical approach, Amiri et al. [29] proposed a modification to the FEM model of the HYBRID III manikin by providing a detailed representation of the lumbar spine. This model was analyzed at a range of frequencies to examine its dynamic behavior. Similarly, Dong et al. [30] developed a computational model of a seated human body using FEM to predict the effects of factors on the biodynamic characteristics of the spine. The model incorporated the skeleton, muscle, viscera, ligament, intervertebral discs, and skin within POSER Pro 2014 software. Their model predicted resonance frequencies between 4 and 7 Hz. Concerning posture effects, the study found that an upright posture promotes even stress distribution, highlighting the importance of maintaining correct posture to reduce the risk of intervertebral disc damage.

Demonstrating the Capability of Artificial Intelligence Methods in Vibration Problems [31]. Zhang et al. [32] developed an Artificial Neural Network (ANN) model optimized by a Genetic Algorithm (GA), called GA-BP-ANN to predict seat transmissibility in an occupant-seat system under WBV exposure. The GA-BP-ANN model showed very high prediction accuracy on the test dataset, with RMSE values between 0.071 and 0.073 and R^2^ values from 0.945 to 0.948. The variables that most influenced the model’s predictability were backrest inclination, vibration magnitude, and cushion thickness.

Building on their previous work, Amiri et al. [33] used the modified computational model with time-varying material properties of the spinal discs in the seated HYBRID III manikin to evaluate the impacts of vehicle vibration on driver’s lumbar discs and their association with spinal problems. They assessed how variations in WBV parameters—such as frequency, seat inclination, and posture—affect spinal response and injury risk. For a driver working 6 h a day for 15 years, the risk of spinal injury was found to be low. Although changing the inclination slightly altered the risk, it remained minimal. The factor with the greatest influence on injury risk was vibration frequencies close to the lumbar spine’s resonance frequency.

Xin et al. [34] developed a nine-degree-of-freedom mathematical model of a “human–crane–rail” system, in accordance with the ISO 2631-1 standard. They applied a particle swarm optimization (PSO) algorithm to optimize the structural design of a 100-ton, 28.5 m double-girder overhead crane. The optimization considered structural parameters, an annoyance rate model, and the crane’s acceleration and displacement amplitudes. The results demonstrated a decrease in the human annoyance rate from 28.3% to 9.8%, and a reduction in the root mean square of the weighted human vibration acceleration from 0.59 m/s^2^ to 0.38 m/s^2^.

To identify vibrations that could potentially induce resonance in crane operators, Silva et al. [35] conducted an analysis using the finite element method with ANSYS software version 2023 r2. Their findings revealed three vibration frequencies—1, 2, and 8 Hz—that may cause resonance in the operator’s head, feet, and torso.

The use of computer models based on the finite element method has been widely used to understand, analyze, and come up with quick solutions to prevent WBV amplitudes that are harmful to users. However, due to the complexity of the human body, the development of models using the FE methodology is simplified to compensate for the reduction in computational effort [36] making it possible to validate the modeling using experimental data.

Table 1 summarizes the most recent work on developing numerical analysis of whole-body vibrations using the finite element methodology.

Based on the state-of-the-art review, it is evident that numerous studies have evaluated WBV across various fields and for diverse purposes. However, investigations focusing on modal and harmonic analysis specifically applied to Portainer operators remain limited in depth. Therefore, this work aims to assess the impact of those container handling machines operation on operator’s exposure to occupational WBV. To achieve this, a 3D model of a seated human was developed, and modal and harmonic analyses were performed using FEM within ANSYS software. Additionally, quantitative WBV measurements were conducted at actual workstations to validate the developed model.

The main contributions of this work are:

- The development of a novel model tailored to the occupational profile of port crane operators, especially those operating Portainers, thereby expanding scientific understanding of WBV effects in this specific context.
- Integration of empirical vibration data with numerical simulations through a computational model based on modal and harmonic analysis via the FEM, enhancing the accuracy and realism of the human-seat dynamic interaction.
- Direct contributions to ergonomics and occupational safety by providing results that support the mitigation of ergonomic risks and offer technical guidance for redesigning seats and cabins, with special attention to the most critical frequency ranges affecting anatomical structures such as the spine.

## 2. Materials and Methods

This section outlines the methodological procedures employed to evaluate WBV exposure among Portainer operators. It includes the mathematical modeling and simulation of the seat-operator system, the experimental measurement of WBV in an actual operational environment, and the development of a computational model using the FEM. Each subsection details a specific stage of the process, progressing from simplified dynamic models to comprehensive 3D simulations.

### 2.1. Mathematical Modeling of Whole-Body Vibration

In this study, a mechanical model of the suspension seat of a vibration isolation system for a machine is presented in one dimension (1D). The model represents the system’s behavior and is used for initial pre-processing to facilitate simulation and processing speed. This provides preliminary results in global analysis. The equations for mass, damping, and stiffness parameters were determined through literature research and initially processed in the Simulink^®^ software version 2016. This tool simulates the vibrations affecting the operators’ bodies and predicts their consequences.

The vibration intensities that the operators of the respective machines are subjected to were measured to validate the model. These values were compared with the levels found in the 3D numerical simulations using the software Ansys Mechanical^®^ Version 2023 r2. The results of the measurements and simulations were then compared to determine whether they are similar or approximate. This was the validation of the model against real data.

**Figure 1:**
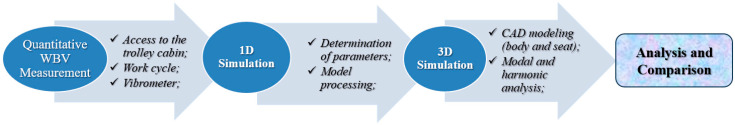
Methodological implementation flowchart.

The study was limited to the workstation of the container operator for feasibility reasons. Figure 1 shows a flowchart of the implementation of the numerical methodology applied in this study.

### 2.2. Quantitative Whole-Body Vibration Assessment

**Figure 2:**
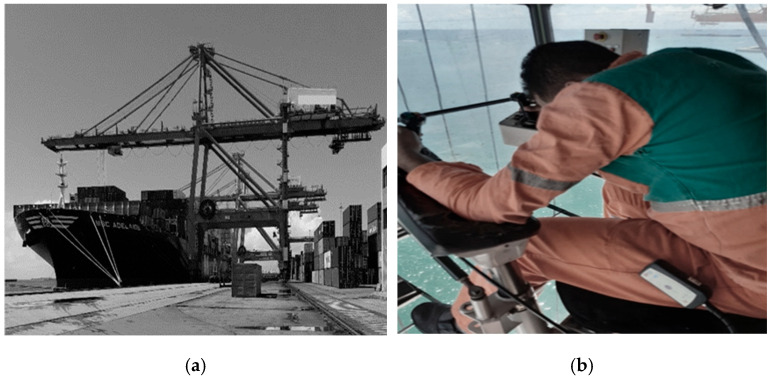
Measurement scheme. (**a**) Portainer. (**b**) Portainer operation [41].

Quantitative measurements of whole-body occupational vibration were conducted between November 2021 and February 2022 at a port terminal in northeastern Brazil. The study focused on occupational exposure to WBV during port container handling operations using the Portainer, responsible for loading and unloading ships (Figure 2a). The study involved quantitative WBV measurements of Portainer operators (Figure 2b) using a vibrometer with the following specifications: operating temperature −10 °C to 50 °C (0–95% RH), 0.01 measurement resolution, resonance frequency greater than 36 kHz, and processing of results in the accompanying software dBA8 dBA8 v1.0.2.8 and dBMaestro. For the complete procedure for measuring and processing the data, please refer to the literature [41].

The assessment was conducted on three of the four operational container quay cranes (Portainers) at the terminal. The terminal has a total of six cranes: two cranes (Portainers 1 and 2) were inactive and therefore not assessed. Four cranes were operational, but measurements were limited to three due to a restriction on the fourth: Portainer 4 could not be measured because its access elevator was out of order. For safety reasons, access to the cabin via the stairs was restricted to the operator present during the assessment.

ISO 2631 defines frequency weightings, which are standardized electronic filters used to measure and evaluate human exposure to whole-body vibration [27]. These filters, such as W_k_, W_d_, and W_f_, are essential because they consider how the human body perceives vibration differently across various frequencies and axes of motion. Each weighting curve is designed for a specific type of vibration to accurately reflect the body’s sensitivity. In this study, the W_k_ and W_d_ weightings were applied. These weightings, as required by ISO 2631, adjust physical vibration measurements to accurately represent how the human body perceives and responds to vibration across different frequencies and axes of motion. The vibrometer used for quantitative assessments was the 01dB VIB, designed for measuring occupational whole-body vibration in compliance with ISO standards, European Directive 2002/44/EC, and ACGIH recommendations. The equipment was calibrated and accompanied by an RBC calibration certificate from the INMETRO-accredited laboratory. This ergonomic instrument is user-friendly, lightweight, and capable of measuring data, processing signals, and transferring stored data. It stores vibration levels on the X, Y, and Z axes, changes resulting from standardized exposure A(8) daily exposure dose and resulting vibration dose value (VDV) using the dB A(8) and dBMaestro software, and records the signal and spectrum in 1/3 octaves. The measurement results were compared with Table 2, which was adapted from the Brazilian technical standard NHO 09 [42].

### 2.3. One-Dimensional Dynamic Modeling

**Figure 3:**
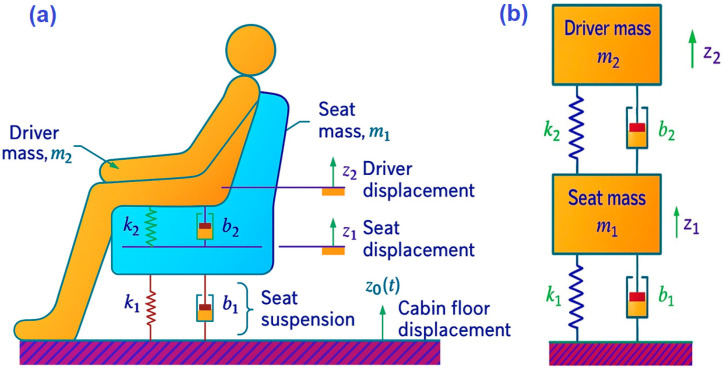
Numerical modeling scheme. (**a**) Schematic Diagram of Seat Suspension System. (**b**) Mechanical Model for Seat Suspension System. Adapted from [43,44].

This section presents the numerical modeling of a suspension seat system based on a one-dimensional dynamic model. The model is based on a vibration isolation system for a vehicle, similar to the Portainer operator’s workstation, with the same parameters. The study emphasizes the working position of machine operators. A preliminary analysis was conducted using a one-dimensional simulation with the schematic diagram proposed by [36] in Figure 3a, which considered parameters such as mass, stiffness, and damping.

Figure 3b shows the concentrated mechanical model of the suspension-seat system. The seat’s total mass is represented by m_1_, while m_2_ represents the driver’s mass. The ideal spring k_1_ and viscous damper b_1_ model is the physical damper, connecting the seat to the vehicle cabin floor. The ideal spring k_2_ and friction coefficient b_2_ represent the stiffness and damping of the seat cushion. Finally, z_1_ and z_2_ are the vertical displacement of the seat mass and driver’s mass, respectively. Both are measured relative to their static equilibrium positions. The cabin floor’s vertical displacement due to vibrations induced by displacement and terrain is represented by z_0_(t), with the upward direction being the positive sign convention for all displacements [43].

**Figure 4:**
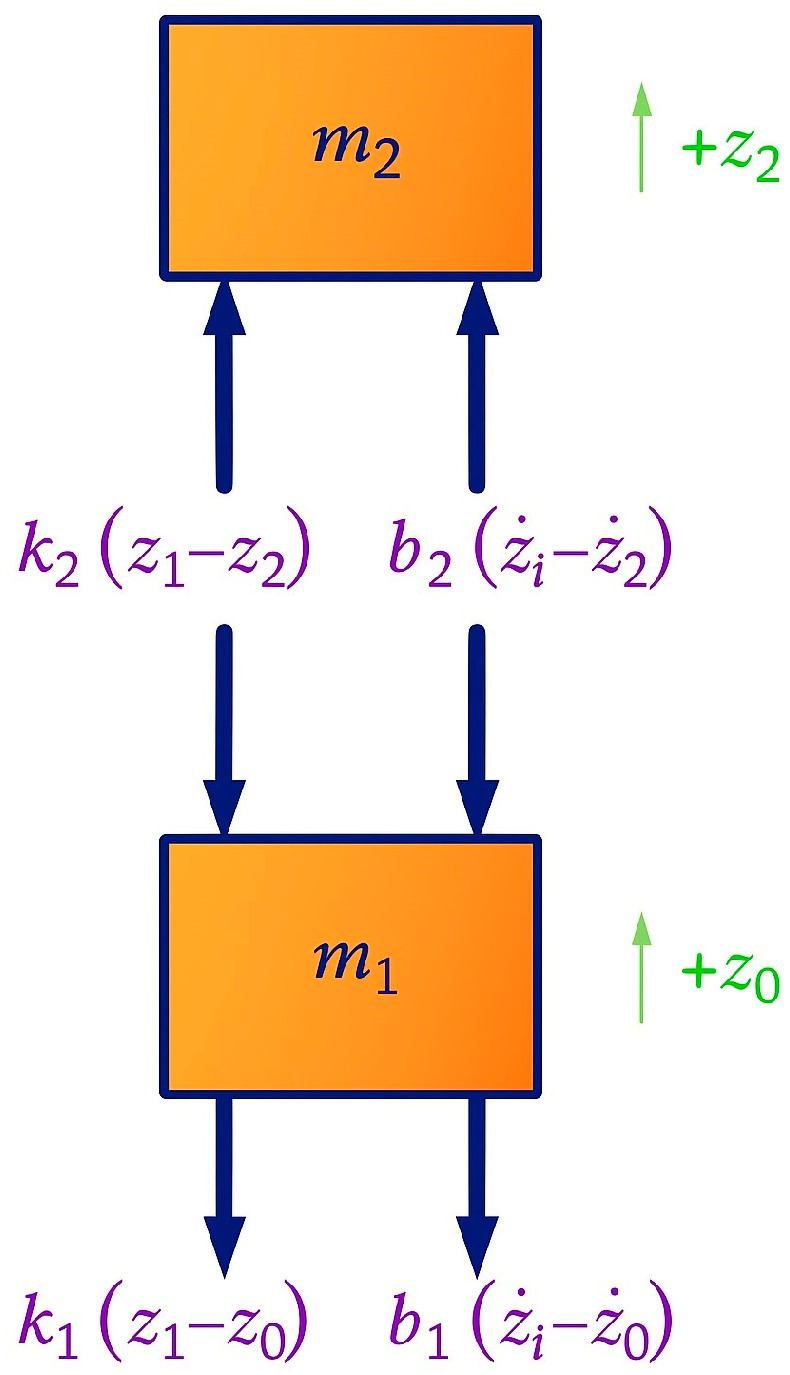
Free body diagram for the seat suspension system. Adapted from [43,44].

In Figure 4, a free-body diagram of a two-mass mechanical system is depicted, with a positive (upward) convention for the displacements z_1_ and z_2_. All spring and damper forces depend on the relative displacements and velocities between the mass of the seat and the cabin floor, and the masses of the seat and the driver, respectively. When the suspension spring k_1_ is in traction and the reaction force acts downwards on the seat mass m_1_, the relative displacement z_1_ − z_0_ is assumed to be positive. Similarly, the relative displacement z_1_ − z_2_ is positive when the seat cushion is compressed, and the reaction force acts downwards on the seat mass m_1_ and upwards on the driver mass m_2,_ as shown by the equal and opposite spring forces k (z_1_ − z_2_) in the free body diagram.

As per the diagram from Kluever [43] and Yang et al. [44], friction forces are dependent on the relative speed. In case the relative velocity z_1_ − z_0_ is positive (i.e., the seat mass m_1_ is moving “outwards” relative to the cabin floor), the friction reaction force b_1_(z_1_ − z_0_) on the mass m_1_ will oppose the relative movement, as shown in the free-body diagram. Similarly, if it is assumed that the relative velocity z_1_ − z_2_ is positive, then the reaction force from the seat cushion damping acts downwards on the seat mass m_1_ and upwards on the driver m_2_. This is demonstrated by the equal and opposite damper forces b_2_(z_1_ − z_2_) in the diagram.

Applying Newton’s second law, we get:

(1) $$Mass1:+\sum F=-k_{2}\left(z_{1}-z_{2}\right)-b_{2}\left(\dot{z_{1}}-\dot{z_{2}}\right)-k_{1}\left(z_{1}-z_{0}\right)-b_{1}\left(\dot{z_{1}}-\dot{z_{0}}\right)=m_{1}{\ddot{z}}_{1}$$

(2) $$Mass2:+\sum F=k_{2}\left(z_{1}-z_{2}\right)+b_{2}\left(\dot{z_{1}}-\dot{z_{2}}\right)=m_{2}{\ddot{z}}_{2}$$

From Equations (1) and (2), and rearranging, comes:

(3) $$m_{1}{\ddot{z}}_{1}+b_{1}\dot{z_{1}}+b_{2}\left(\dot{z_{1}}-\dot{z_{2}}\right)+k_{1}z_{1}+k_{2}\left(z_{1}-z_{2}\right)=b_{1}{\dot{z}}_{0}\left(t\right)+k_{1}z_{0}\left(t\right)$$

(4) $$m_{2}{\ddot{z}}_{2}+b_{2}\left(\dot{z_{2}}-\dot{z_{1}}\right)+k_{2}\left(z_{2}-z_{1}\right)=0$$

Putting Equations (3) and (4) together, and considering $x_{1}=z_{1,}x_{2}={\dot{z}}_{1},x_{3}=z_{2},x_{4}={\dot{z}}_{2}$ as state variables, and $z_{0}\left(t\right)=u_{1}$ and ${\dot{z}}_{0}\left(t\right)=u_{2}$ as input variables, in matrix form, using Equation (5):

(5) $$\left[\begin{array}{c} {\dot{x}}_{1}\\ {\dot{x}}_{2}\\ \dot{x_{3}}\\ {\dot{x}}_{4} \end{array}\right]=\;\left[\begin{array}{cccc} 0 & 1 & 0 & 0\\ \frac{{-(k}_{1}+k_{2)}}{m_{1}} & \frac{{-(b}_{1}+b_{2)}}{m_{1}} & \frac{k_{2}}{m_{1}} & \frac{b_{2}}{m_{1}}\\ 0 & 0 & 0 & 1\\ \frac{k_{2}}{m_{2}} & \frac{b_{2}}{m_{2}} & -\frac{k_{2}}{m_{2}} & -\frac{b_{2}}{m_{2}} \end{array}\right]\left[\begin{array}{c} x_{1}\\ x_{2}\\ x_{3}\\ x_{4} \end{array}\right]+\left[\begin{array}{cc} 0 & 0\\ \frac{k_{1}}{m_{1}} & \frac{b_{1}}{m_{1}}\\ 0 & 0\\ 0 & 0 \end{array}\right]\left[\begin{array}{c} u_{1}\\ u_{2} \end{array}\right]$$

The two system outputs or measurements were specified as y_1_ = z_2_ e y_2_ = $\ddot{z}$_2_. The first output equation is simply y_1_ = x_3_ and the second is obtained from the terms of the last line in Equation (5).

(6) $$\left[\begin{array}{c} y_{1}\\ y_{2} \end{array}\right]=\left[\begin{array}{cccc} 0 & 0 & 1 & 0\\ \frac{k_{2}}{m_{2}} & \frac{b_{2}}{m_{2}} & \frac{-k_{2}}{m_{2}} & \frac{-b_{2}}{m_{2}} \end{array}\right]\left[\begin{array}{c} x_{1}\\ x_{2}\\ x_{3}\\ x_{4} \end{array}\right]+\left[\begin{array}{cc} 0 & 0\\ 0 & 0 \end{array}\right]\left[\begin{array}{c} u_{1}\\ u_{2} \end{array}\right]$$

The mass, stiffness, and damping data required to process the 1D model shown in Table 3 were taken from related works [45,46].

The model was developed using the MatLab Simulink computer platform. Simulations of mass displacement and driver acceleration over time were performed based on the model proposed in the literature by Amiri et al. [29,33].

In the simulation, the weight of the crane seat was set to 62 kg, as specified in the TER brochure [50]. This decision was based on significant variations in the weight of the seat reported in the literature, influenced by the types of machines studied, including trucks, tractors, and graders. The weight of the operator was set at 84 kg, reflecting the average mass of terminal port container operators.

To assess the response of the suspension-seat system, the simulations were conducted with various inputs. Initially, a “peak” in the displacement of the cabin floor z_0_(t) was considered. This was modeled as a “triangular pulse” with a peak of 0.03 m (3 cm) and a constant vertical “lift rate” of ż_0_ = 5.4 m/s (upward). The total pulse duration was 5.6 ms.

The system was configured to analyze the driver’s displacement and acceleration as functions of time and transmissibility. The latter represents the ratio between the input and output displacements.

### 2.4. Computational Model Using the Finite Element Method

A three-dimensional model was created to simulate the workstation of a Portainer operator. The goal of this simulation was to predict the operating behavior of a man-seat system through modal and harmonic analysis using the finite element method (FEM), with the ANSYS^®^ 2022 R2 simulation tool. The simulation aimed to determine the natural vibration frequency of the model based on its mass distribution and stiffness characteristics.

To simulate the transmission of vibrations to the operator, an external vibration load was applied from equipment sources. The simulation generated acceleration and displacement results as a function of frequency, which were compared to the model developed with the results of quantitative measurements taken *in loco* on the Portainer.

The three-dimensional model was built in the following phases: CAD modeling and simplifications, processing settings, the properties of the Portainer seat, simulation parameters, mesh parameters, and boundary conditions.

The coordinate system adopted aligns the *Y*-axis with gravity acceleration, pointing upwards from the floor, whereas *X*-axis aligns with the line connecting the shoulders of the human model, positive to its left side. Consequently, the *Z*-axis is normal to the human body’s chest, pointing outwards.

#### 2.4.1. CAD Modeling and Its Simplifications

The crane seat was modeled at full scale using SPACECLIM CAD software version 2023 r2, which is a part of the ANSYS^®^ simulation platform. The design was based on the literature [51], specifically developed for Portainer operators.

**Figure 5:**
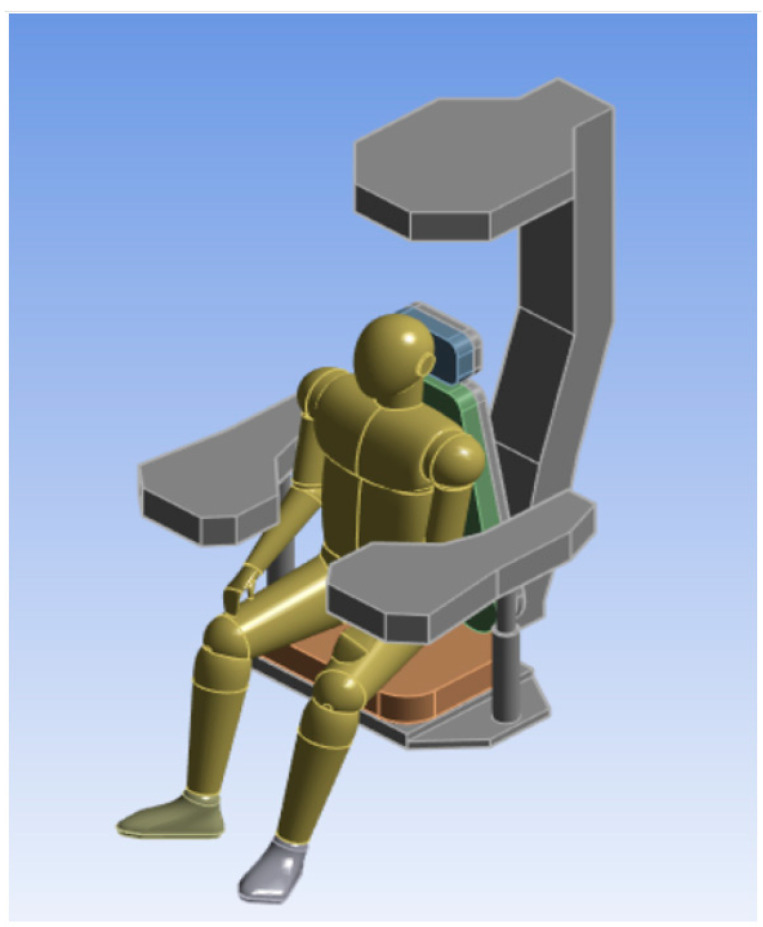
Model developed for the Man-Seat System [35,41].

The seat frame is constructed using ASTM A36 structural steel, which is 3 mm thick and has a density of D = 7.85 × 10^3^ kg/m^3^. It has a Young’s modulus of *E*= 2 × 10^5^ MPa and a Poisson coefficient of ν = 0.26. The seat and backrest foam are made of polyurethane with a density of D = 12.66 Kg/m^3^, Young modulus *E* = 25 MPa, and Poisson coefficient ν = 0.30 [52]. Figure 5 shows the finished model of the seat that meets the specified requirements, with a total weight of 109.30 kg. For simulation purposes, the seat model was fixed at the seat structure solidary with the seat.

The human body used was created in 3D using the Solidworks^®^ platform, as illustrated in Figure 5, and as it supported by literature [35,41]. It was represented as a homogeneous and isotropic solid, meaning its material properties are uniform in all directions. The model has a density of D = 1062 kg/m^3^, a Young’s modulus of 13 MPa, and a Poisson’s ratio of ν = 0.30 [40,53].

#### 2.4.2. Simulation Processing Settings

The interaction between the human body and the seat occurs primarily at three contact points: the headrest, the seat base, and the backrest. Specifically, the headrest supports the head, the seat base interfaces with the thighs and buttocks, and the backrest engages the thoracic and cervical regions of the spine [35,41]. To ensure optimal mesh selection for the computational model, a thorough analysis was performed, comparing three distinct mesh configurations, as summarized in Table 4 [35,41].

**Figure 6:**
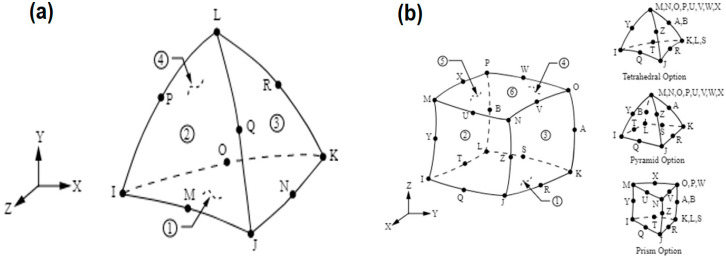
Analyzed mesh. (**a**) 3D Tetrahedric mesh. (**b**) 3D Hexahedric mesh [54].

The element SOLID187 consists of ten higher-order nodes in a 3D space, Figure 6a. It is characterized by quadratic displacement behavior and is best suited for modeling irregular meshes that lack symmetry. The element has ten nodes, and each node with three degrees of freedom in the X, Y, and Z-directions. The element also possesses several properties such as plasticity, hyper elasticity, creep, tension stiffening, and the capacity to undergo large deflection and deformation.

The SOLID186 is a 20-node, higher-order 3D solid element that exhibits a quadratic displacement behavior. It consists of twenty nodes with three degrees of freedom each in the X, Y, and Z nodal directions. The element has several properties such as plasticity, hyper elasticity, creep, tension stiffening, large deflection, and the capacity to undergo large deflection and deformation. It is illustrated in Figure 6b.

Tetrahedral and hexahedral elements were selected for the model due to their ability to accurately represent complex structures [35,41], such as the human body [52,55,56]. The simulations were conducted on a computer equipped with an Intel^®^ Xeon W-1250 processor at 3.30 GHz and 16.00 GB of RAM (15.7 GB usable) [35,41].

## 3. Results

This section presents the results of the models developed for the WBV study. The following subsections address:

- Quantitative assessment of Whole-Body Vibration (WBV).
- Evaluation of the one-dimensional model simulation (1D).
- Evaluation of the three-dimensional model simulation (3D).

### 3.1. Model Comparison—Whole-Body Vibration

A comparison between the numerical and experimental values was conducted focusing on seat acceleration. The goal was to validate the model’s predictions within the frequency range of 2 to 4 Hz, as shown in Table 5 [35]. The accelerations were analyzed at a corresponding point on both the portainer operator’s seat and on the model’s seat, as shown in Figure 2b. A detailed explanation of this validation is provided in the previous work by Silva et al. [35].

Table 5 compares the measured acceleration of the portainer operator’s seat with the acceleration predicted by model at three frequencies. At 2 Hz, 3 Hz, and 4 Hz, the acceleration values are all of the same order of magnitude (10 − 2 m/s^2^). These comparative results show that the model generally captures the system’s dynamics, supporting the validity of the modeling approach. To validate the comparison, vibration magnitudes were correlated. Notably, the lowest relative error is 10.00% at 3 Hz, corresponding to a difference of 0.01 m/s^2^. The 3 Hz error is three times smaller than the 33.00% error at 2 Hz, indicating significantly better performance at 3 Hz. Although the error at 4 Hz is 18.18%, which is still better than at 2 Hz, it is only about 1.8 times smaller, not three times. The reduced error at 3 Hz and 4 Hz compared to 2 Hz together demonstrate that the model performs better at the higher frequencies investigated [35].

### 3.2. Quantitative Assessment of Whole-Body Vibration

Quantitative measurements were taken to assess whole-body vibration experienced by 12 Portainer operators, according to NHO 09 standards [42]. The study evaluated the accelerations of resulting normalized exposure A(8) and the resulting vibration dose value (VDV), which were then compared with the tolerance limits [42] summarized in Table 2.

**Figure 7:**
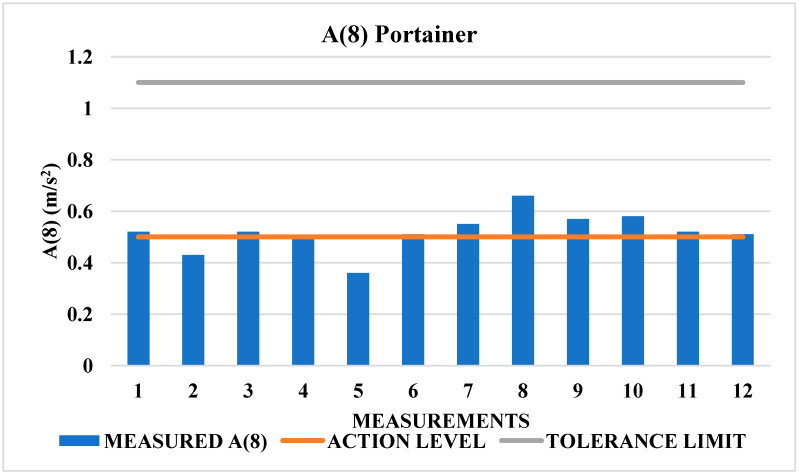
Vibrational behavior of A(8) Portainer values.

Table 6 indicates that none of the Portainer operators exceeded the A(8) tolerance limit of 1.1 m/s^2^ for whole-body vibration (WBV). However, 75% of the total measurements taken exceeded the action level 0.5 m/s^2^, as shown in Figure 7.

In these situations, where the vibration effects exceed the action limit, the thresholds in Table 2 suggest the adoption of preventive measures as prescribed in NHO-09. If the resulting vibration dose values (VDV) exceed the action level, systematic control actions must be implemented to minimize the likelihood of occupational exposures exceeding the exposure limits, as per NHO-09 [42].

**Figure 8:**
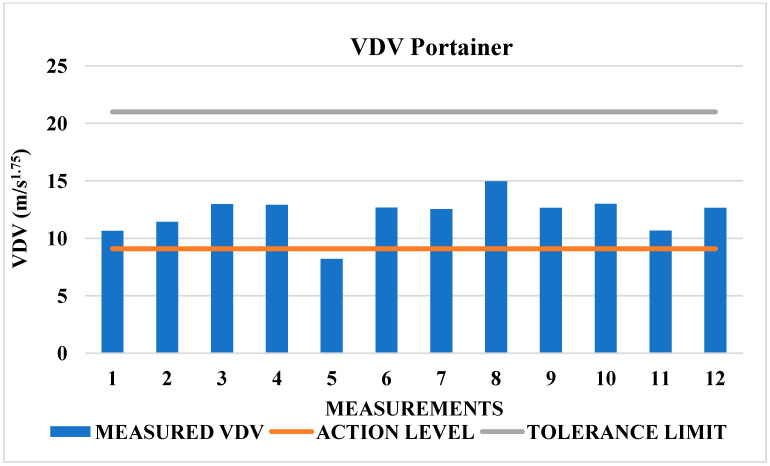
Vibrational behavior of VDV Portainer.

Although the tolerance limit 21 m/s^1.75^ was not exceeded in this case, 92% of the total measurements exceeded the action level 9.1 m/s^1.75^, as shown in Figure 8. Therefore, preventive measures must be adopted as per the thresholds in Table 2, and systematic control actions must be implemented as per NR-09 [42].

**Figure 9:**
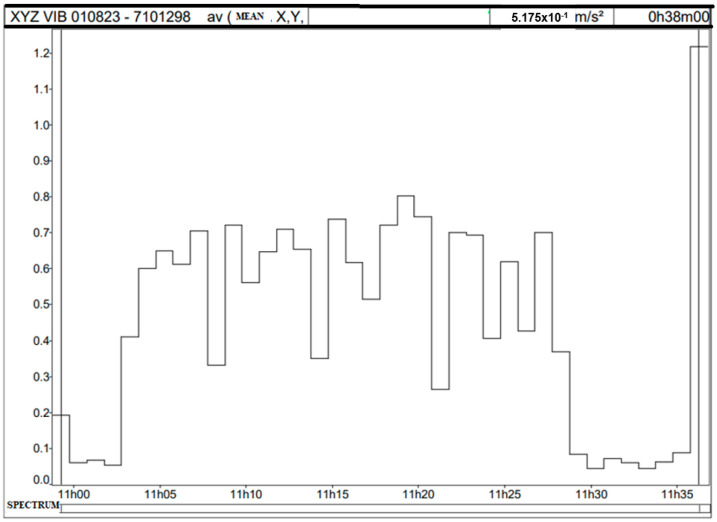
Average resultant acceleration in the X, Y, and Z axes over time.

**Figure 10:**
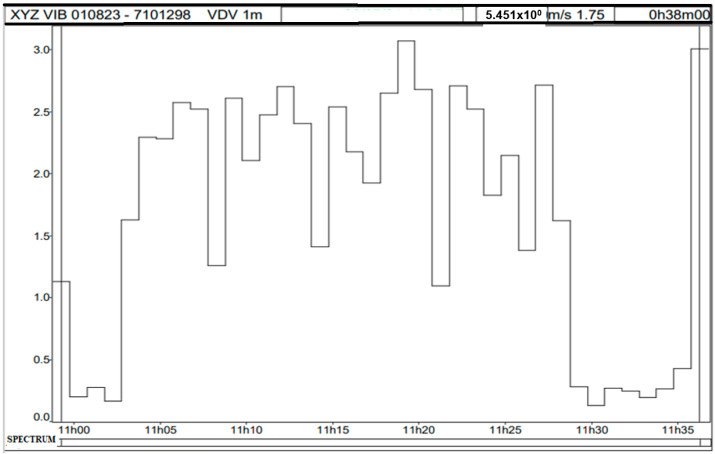
Vibration dose on the X, Y, and Z axes in time.

Figure 9 and Figure 10 present the analysis of acceleration data recorded during a measurement on the Portainer. The measurement yielded an A(8) value of 0.52 m/s^2^ and VDV of 10.64 m/s^1.75^. The data were collected over an average period of 30 min. The analysis was performed using DbMaestro software dBA8 v1.0.2.8. Figure 9 shows the average acceleration along the three axes throughout the measurement period, with a peak of approximately 0.8 m/s^2^ occurring around 11:19 a.m. The overall average acceleration during this time was 0.517 m/s^2^.

Figure 10 displays the instantaneous vibration dose values (VDV) on the three axes at different time points during the measurement. The total VDV recorded during the measurement period was 5.45 m/s^1.75^, normalized for an eight-hour working day, resulting in VDV = 10.64 m/s^1.75^.

**Figure 11:**
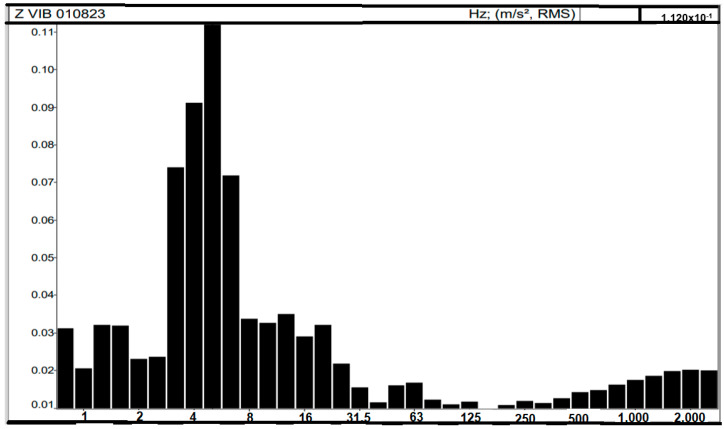
Frequency distribution with acceleration on axis Z.

Figure 11 shows the frequency distribution in hertz (Hz) compared to the instantaneous acceleration on the *Z*-axis. The most predominant frequency in the spectrum is 5 Hz, followed by 3 and 4 Hz.

According to Wasserman [57], when sitting, the body’s resonance frequencies in the *Z*-axis can fluctuate between 3 and 5 Hz on the spine and from 2 to 6 Hz on the shoulders.

**Figure 12:**
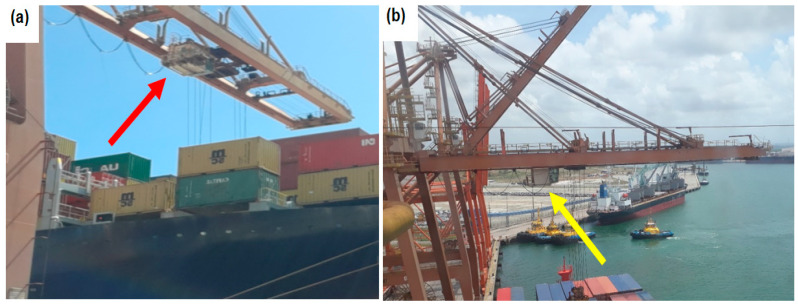
(**a**) Position of the Portainer cabin. (**b**) Luffing jib of quay crane.

In the Portainer, the operating cabin (trolley) slides on rails powered by an electric motor, as shown in Figure 12a. The rails and the lifting crane joints experience the highest incidence of shocks. Figure 12b displays two Portainer cranes: one raised for ship docking and the other in the operating position. The joint we refer to is where the crane articulates, which sometimes becomes misaligned with the rest of the structure. When the trolley passes over this joint, it experiences a stronger impact, increasing shocks and the vibration dose value (VDV). These impacts are visible in Figure 8, which highlights the resulting peaks in VDV.

### 3.3. Evaluation of the One-Dimensional Mode Simulation (1D)

This section presents the results of the simulation tests performed using the one-dimensional (1D) model. Firstly, the results of the 1D simulations conducted in the Simulink^®^ software are presented, using stiffness, damping, and mass data from Table 3.

The processing of the simulations generated three types of charts. The first chart shows the displacement behavior of the driver’s mass over time in millimeters. The second illustrates the driver’s acceleration over time in meters per second squared. Lastly, the hird chart displays transmissibility based on frequency. Transmissibility is the ratio between the output displacements and the input displacement. In the suspension-seat system shown in Figure 4, the driver displacement z_2_ and the floor displacement z_0_ represent the output and input displacements, respectively. The transmissibility analysis was carried out using the following criteria:

- When transmissibility is equal to 1, it means that the displacement on the floor of the machine or vehicle is completely transmitted to the driver, causing the driver to also move by the same amount.
- When the driver moves more than the floor it means that the transmissibility is greater than 1 and the system has boosted the displacement of the floor.
- When the driver moves less than the floor, the transmissibility is less than 1, which means that the system has damped the displacement of the floor.

Table 7 shows 5 combinations of stiffness, damping, and mass data. The parameters of the mechanical system are represented by the letters “m”, “k” and “b”.

m_1_ = seat mass, kg;

m_2_ = driver’s mass, kg;

k_1_ = suspension stiffness, N/m;

k_2_ = seat cushion stiffness, N/m;

b_1_ = suspension friction, Ns/m;

b_2_ = seat cushion friction, Ns/m.

**Figure 13:**
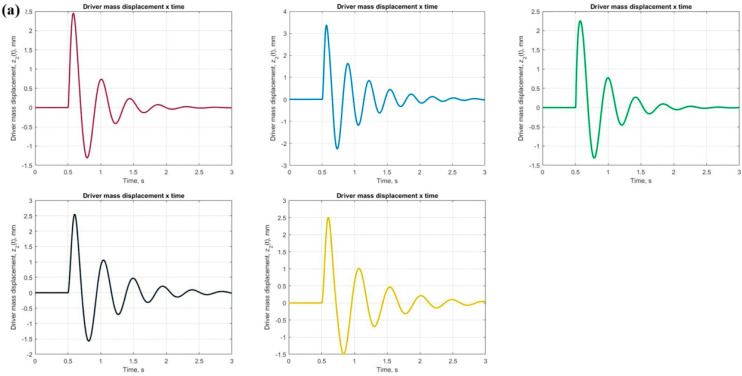

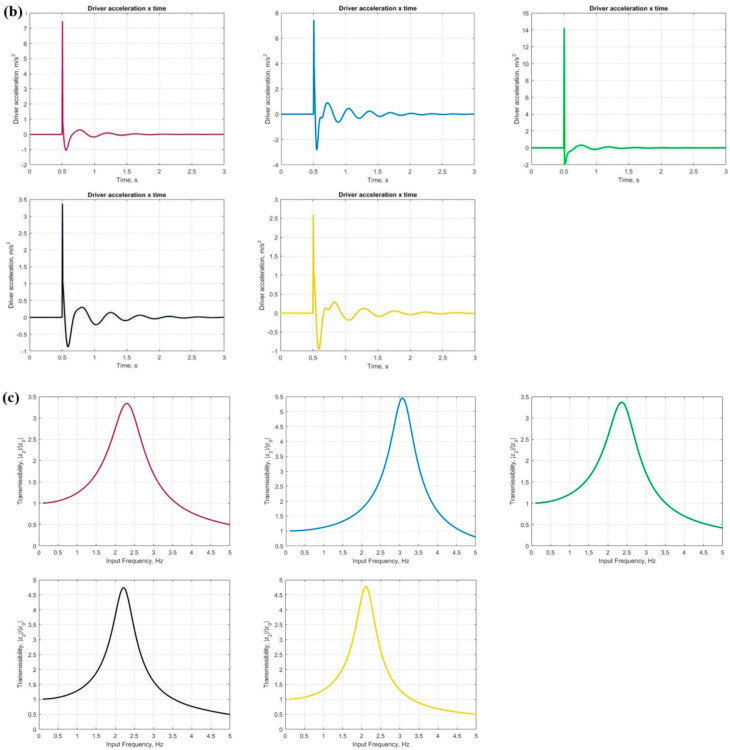
Results of Cases 1–5. (**a**) Displacement x time. (**b**) Acceleration x time. (**c**) Transmissibility X Frequency.

Case 1 utilized data from Bolina [47] supplemented by additional sources found in the literature [45,46] as well as the median operator weight (m_2_ = 84) of the interviewed subjects. Figure 13a illustrates a displacement of approximately 2.5 mm in the driver’s mass after 0.5 s of the pulse, which then decreases to around 1.5 mm on the opposite side and gradually diminishes due to damping. Figure 13b also depicts the driver’s acceleration over time, reaching 7.5 m/s^2^ at 0.5 s before rapidly decelerating to zero. Additionally, Figure 13c presents transmissibility as a function of frequency, with a peak value of 3.4 occurring in the 2 to 2.5 Hz range.

In Case 2 data from Anflor et al. [48] was used together with data from [49]. Figure 13a shows the displacement of the driver’s mass, which reaches 3.5 mm initially, followed by a reduction to 2.3 mm in the opposite direction, then 1.5 mm in the original direction, before gradually damping while oscillating toward zero. The driver’s acceleration over time is also shown in Figure 13b, peaking at 7.5 m/s^2^ during the pulse application. Additionally, Figure 13c displays a peak transmissibility of 5.5 occurring near 3 Hz.

In case 3, data from Zhao et al. [46] was combined with that from Yin et al. [45]. The results indicate a peak driver displacement of 2.3 mm with subsequent damping, as illustrated in Figure 13. An acceleration slightly above 14 m/s^2^ was recorded within 0.5 s from the start of measurement, which then decreases to zero, as shown in the same figure. The transmissibility is enhanced, reaching a peak of 3.3 at approximately 2.3 Hz.

In Case 4 data from Yin et al. [45] was combined with that from Bolina [47], along with the seat weight m_1_ = 62 kg taken from the TER leaflet [50]. Figure 13 presents a maximum peak displacement of 2.5 mm for the driver, moving to 1.5 mm in the opposite direction within the first second, followed by damping oscillations toward zero. The driver’s acceleration reached 3.4 m/s^2^ within 0.5 s and then diminished to zero, as shown in Figure 13b. Transmissibility peaks between 2 and 2.5 Hz, reaching a value of 4.7.

Finally, in Case 5, the parameters from Wei and Griffin [49] were applied, using the median operator mass m_2_ = 84 kg from those interviewed in this study, and a seat mass m_1_ = 64 kg. The results, shown in Figure 13, indicate that the driver’s displacement decreased from 2.5 mm to 1.5 mm toward the opposite side within the first second, then gradually damped until the displacement reached zero. The driver’s acceleration peaked at 2.6 m/s^2^ after 0.5 s and then dropped to zero, as shown in Figure 13b. The transmissibility reached 4.75 between 2 and 2.2 Hz, also displayed in Figure 13c.

**Figure 14:**
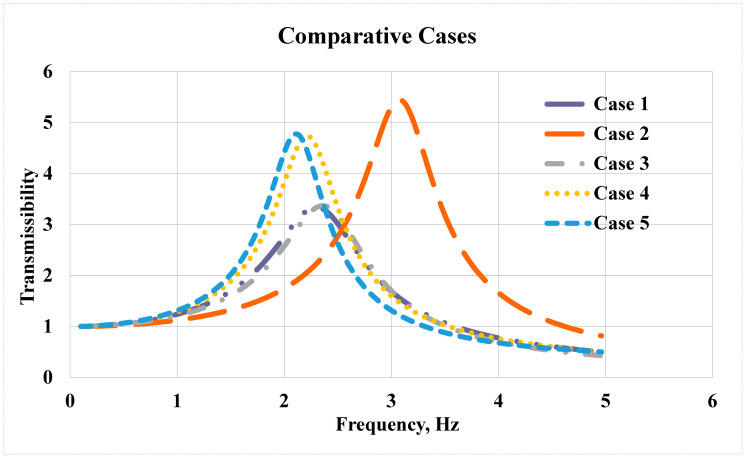
Comparative analysis of transmissibility Cases 1, 2, 3, 4 and 5.

A comparative analysis of the transmissibility of the cases was carried out across Cases from 1 to 5, as presented in Figure 14. Comparing Cases 1 and 2 revealed that Case 2 exhibited higher transmissibility. This difference is likely due to Case 2’s suspension having lower damping (friction) and the driver having less mass, resulting in greater vibration transmission to the driver. Specifically, suspension friction in Case 1 was 62.5% higher than in Case 2, which increased the damping factor and consequently reduced the resonance peak.

The comparison between Cases 3 and 4, showed that their parameters were very similar, except for one key difference: the seat mass. Case 3 had a significantly lower seat mass than Case 4, leading to reduced transmissibility. It was also found that the combined effect of driver mass can substantially influence transmissibility, either increasing or decreasing it. Larger differences between these masses amplify this effect.

Finally, when comparing Cases 4 and 5, the transmissibility-frequency graphs display a strong similarity in the behavior of the suspension-seat system. This is attributed to the close proximity of parameter values in both cases.

Based on the comparative analysis of transmissibility in Cases 1 to 5, Cases 1 and 3 showed the best reduction in transmissibility, both with an approximate value of 3.4. Case 1 was considered the optimal configuration because it achieved this reduction with a higher weight than Case 3. Notably, the best reduction in transmissibility corresponded to the most effective friction (damping) rates. The “best friction (damping) rate values” refer to the higher damping coefficients in the optimal configurations—such as Case 1—that effectively absorbed and diminished the incoming vibration, resulting in the lowest transmissibility value (around 3.4). These friction values provided the greatest dissipation of vibrational energy.

### 3.4. Evaluation of the Three-Dimensional Model Simulation (3D)

This section presents the results of simulation tests conducted in three dimensions (3D). Modal analysis evaluations were carried out initially for the three mesh cases to identify the natural vibration modes of the model. The frequency range of 1 to 20 Hz is where whole-body vibrations reach the spine [58].

Wasserman [57] stated that when sitting in a vertical position, at the vertical *Z*-axis, the resonance frequencies vary between 3 and 5 Hz. Iida [59] reported that the natural resonance frequencies of the spine in the vertical direction are between 4 and 8 Hz. Mansfield [60] reported that the resonance frequencies of conventional seats are around 4 to 6 Hz and they provide isolation when loaded by an adult. Based on this frequency range information, the first five vibration modes generated in the three simulation cases were selected, which is presented in Section 3.3.

One of the common elements among the three generated meshes was identified for analyzing the results of deformation and equivalent stress. The aim was to compare the variations in these parameters and determine one of the meshes as the standard for further studies. According to Iida [59], the thigh and buttock region experience the greatest pressure points against the seat in the sitting position, particularly in the bony area of the pelvis known as the ischial tuberosities. In this region, NHO 09 [42] recommends placing the accelerometer for a quantitative assessment of whole-body vibration in the sitting position. Based on these recommendations, a vibration mode and an element were selected in this specific region for deformation analysis.

**Figure 15:**
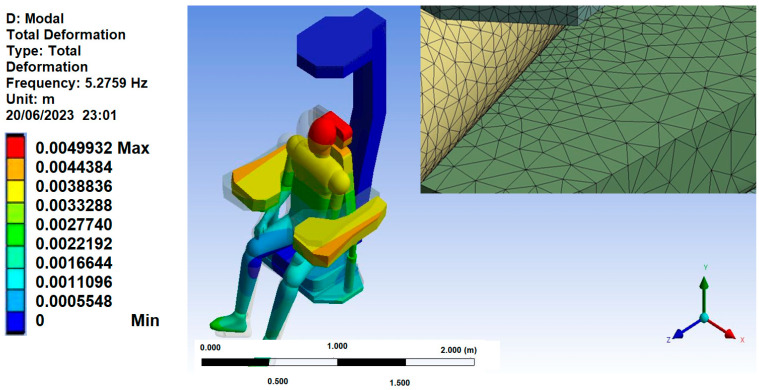
Mesh 1 First Vibration Mode.

**Figure 16:**
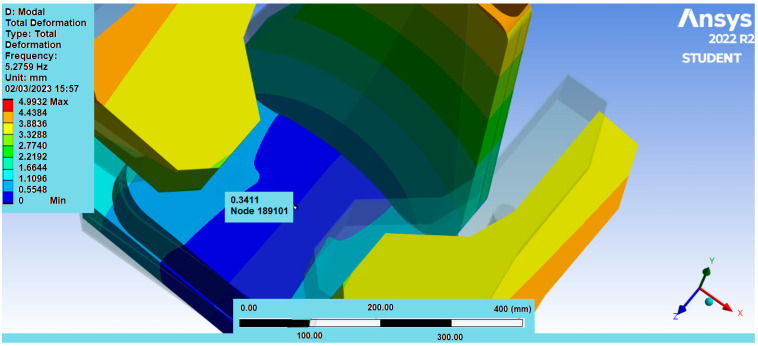
Foam Displacement Region in Mesh 1.

For mesh 1, the initial vibration mode was picked at a frequency of 5.27 Hz, which can be observed in Figure 15. Next, a specific region of displacement was identified in the seat foam, where the seat and the ischial tuberosities of the human model meet, as illustrated in Figure 16. The displacement at this point on mesh 1, was approximately 0.34 mm.

In mesh 2, the first vibration mode was selected similarly, with a frequency of 5.40 Hz. A point identified in the same displacement region as in mesh 1, specifically where the seat contacts the ischial tuberosities of the human model. At this point in mesh 2, the displacement was 0.36 mm.

For mesh 3, the first vibration mode with a frequency of 5.36 Hz was chosen in the same manner as for mesh 1. The point where the seat meets the ischial tuberosities was located in the same deformation region as in mesh 1. In mesh 3, the displacement at this point was also 0.36 mm.

The reference mesh for subsequent studies was selected based on the displacement criterion in the seat foam. If the displacement in the current mesh is equal to or less than 5% compared to the previous mesh, it indicates a stable displacement trend. In such cases, further mesh refinement is unnecessary, as it would significantly increase complexity and processing time.

Table 8 shows the percentage comparison of the displacements for meshes 1, 2, and 3. The percentage difference between meshes 2 and 3 is quite small (0.59%). Therefore, the conclusion is that mesh 2 can be considered the standard mesh for further studies as it displays a tendency towards stability in the displacements.

#### 3.4.1. Modal and Harmonic Analysis

This section presents the results of the modal and harmonic analyses of the studied model. The goal is to determine the natural vibration frequencies of the model and identify its vibration modes based on its mass, stiffness, and damping properties. In the previous section, a mesh analysis was conducted, and the optimal configuration was found to be mesh 2, with 414,210 elements. The first five natural frequencies of this model, used in further analyses, are presented below.

##### Modal Analysis

**Figure 17:**
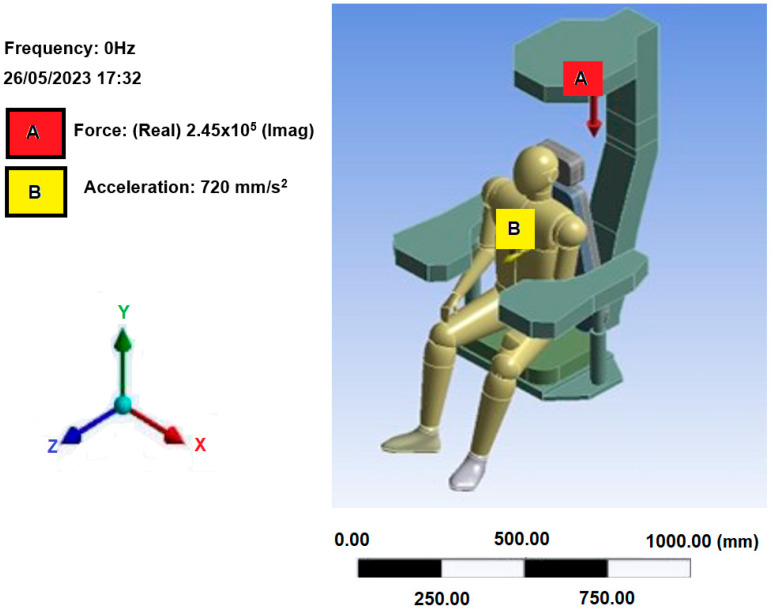
Model with processing configurations.

The study aimed to analyze six vibration modes ranging from 5.40 to 13.06 Hz, related to the trolley motor frequency, which should not exceed 30 Hz [35,61]. According to the literature [57,58,59], the focus was on the occupational vibration frequencies typical for this type of work, particularly around 3 to 8 Hz. A detailed analysis of these modes was previously presented by Silva et al. [35]. That earlier work examined six resonant vibration modes and their effects on the human body. The first mode (5.40 Hz) and second mode (8.10 Hz) primarily affect the head, causing lateral movements along the X and Z axes, respectively. The orientation of these axes is illustrated in Figure 17. The third (9.41 Hz), fourth (9.49 Hz), and fifth (9.97 Hz) modes mainly influence seat supports, with moderate effects on the head and torso. The sixth mode (13.06 Hz) strongly affects the lower limbs, producing significant displacements in the legs and feet [35].

Finite element modal analysis was used to study the modal participation factor, which indicates the amount of mass moving in each direction for each mode. This factor helps to determine how much each mode responds along different axes. Table 9 shows the modal participation factors by mode and axis. The highest modal participation factor occurs along the *X*-axis in the first vibration mode.

##### Harmonic Analysis

To analyze a system under forced vibration and discover its amplitudes, it is necessary to use harmonic analysis, which determines these amplitudes in the frequency domain, thus establishing the system’s frequency response function (FRF).

Bharadwaj and Prakash [53] explain that harmonic analysis helps determine the dynamic behavior of a structure or body under steady-state sinusoidal excitation. This analysis is performed in the frequency domain and is known as frequency response analysis. For instance, during this analysis, the human body model may experience varying acceleration magnitudes within a specific frequency range. The results are expressed as deformation and acceleration responses of the structure, especially in the seat region, shown as acceleration versus frequency, and deformation versus frequency.

By applying appropriate boundary conditions and using the material properties, harmonic analysis was performed on the seat-man model, Figure 17. The system was subjected to a load of 245 kN on the *Y*-axis in a downward direction and an acceleration of 1 m/s^2^ on the *X*-axis. This data was taken from a technical report on the structural calculation of the Portainer at the port terminal [61].

The damping ratio was calculated according to Equation (7):

(7) $$\zeta =b/2{\left(mk\right)}^{1/2}$$

where *b* and *k* are, respectively, the damping and stiffness between the seat and the floor, and m is the mass of the man-seat system. The damping and stiffness data are from [38] as presented in Table 7. To summarize, the boundary conditions used in the harmonic analysis are shown in Table 10.

Table 11 shows the deformation and acceleration results of the model considering a frequency range of 0 to 10 Hz.

**Figure 18:**
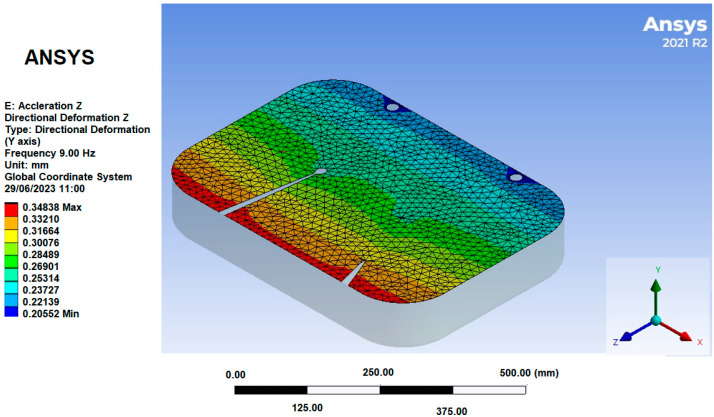
Seat region selected for analysis.

In Figure 18, the seat was the only component extracted, and the region was selected for analyzing deformation and acceleration. The chosen point, which has a deformation of 0.348 mm at 9 Hz, is the region where the operator touches the seat.

**Figure 19:**
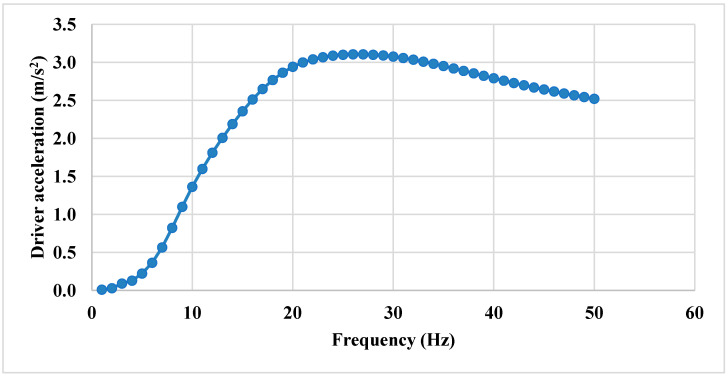
Frequency vs. acceleration profile.

In the frequency range of 1 to 25 Hz, a progressive increase in random acceleration is observed, corresponding to the rising frequency, reaching a peak value of 3.1 m/s^2^, as illustrated in Figure 19. However, beyond 25 Hz, the acceleration starts decaying. As previously reported, Silva et al. [35] conducted a harmonic analysis under a different scenario. The results showed a similar pattern to those identified in the present study.

**Figure 20:**
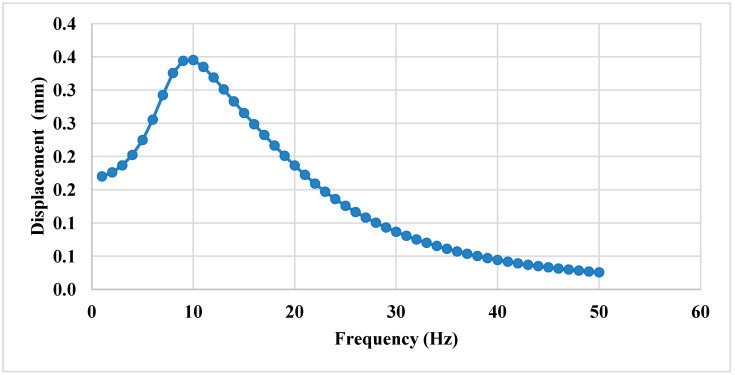
Frequency vs. displacement profile.

The displacement values gradually increase, reaching a maximum of 0.34497 mm at 10 Hz within the analyzed frequency range. Beyond 10 Hz, the displacement values progressively decrease, as shown in Figure 20.

These findings, along with those reported by Silva et al. [35] may prove valuable for future research aimed at establishing parameters to enhance control in the design and manufacturing of machine cabins and seating systems. Computational simulations can be employed during the design phase to identify frequency ranges that may be harmful to human health, thereby aiding the development of safer and more ergonomically sound products.

The results concerning WBVs have revealed extrapolated alert levels, highlighting the need for interventions to improve operating conditions. Similar studies show that A(8) and VDVs were exceeded in dump truck and tractor operators [62,63], although the values remained below tolerance limits. In a study by Campbell [64], which evaluated WBV exposure in crane operators it was concluded that A(8) did not exceed action levels or tolerance limits. However, some VDV results reached the action level, while remaining below the tolerance limit. Therefore, it was concluded that vibration exposure is borderline and requires continuous monitoring of intensity and operator health for Portainer operators.

This study highlights the main challenges faced by Portainer operators and their significant impact on occupational health. Despite modernization efforts, such as the A(8) initiative aimed at improving working conditions, similar problems persist in ports worldwide. Operators continue to endure demanding physical tasks, including climbing increasingly tall Portainer without elevators, prolonged exposure to whole-body vibrations, limited access to sanitary facilities, inadequate equipment maintenance, and the need to work in ergonomically harmful postures.

## 4. Discussion

The results of this study show that 92% of the evaluated operators exceeded the vibration dose value (VDV) action level, although none surpassed the daily exposure tolerance limit A(8). This indicates a borderline condition that requires continuous monitoring, consistent with previous research by [62,64]. Validation of the three-dimensional model with actual data revealed a maximum relative error of 33%; however, this value decreased to 10% at a frequency of 4 Hz, demonstrating higher accuracy precisely within the critical resonance range of the human body (between 3 and 5 Hz), as reported in the literature [35,57,59]. Similarly, Singh et al. [24], focusing on tractors, applied neural networks combined with piezoelectric insulators to mitigate the effects of WBV. They observed similar dominant frequencies, between 4 and 6 Hz, supporting the effectiveness of optimization-based approaches. In other context, Adam et al. [21] identified maximum transmissibility between 1.75 and 2.5 Hz in tractor operators, emphasizing the influence of occupational posture and seat damping characteristics.

Regarding numerical modeling, Gao et al. [37] developed a finite element method (FEM) model with satisfactory response below 10 Hz, compatible with the modal analysis range used in this work. One-dimensional simulations allowed exploration of parametric variations, such as mass, stiffness, and damping, showing that configurations with higher damping and appropriated mass lead to lower transmissibility, highlighting the importance of mechanical seat design.

Additionally, three-dimensional simulations revealed vibration modes with significant activity along the X and Z axes, especially in critical body regions such as the head and lumbar area. The structural deformations observed in the seat, ranging from 0.34 to 0.36 mm, fall within the expected range and agree with findings in the literature [30,64].

A main limitation of the study is the modeling of the human body as a homogeneous and isotropic solid. While computationally efficient, this approach does not accurately represent the complex biomechanical structure of the body, particularly the differences between soft tissues, bones, and intervertebral discs. Therefore, it is recommended to develop more complex and realistic biomechanical models that incorporate differentiated viscoelastic properties for bones, muscles, discs, and ligaments, as suggested in the literature [29,30]. Furthermore, integrating artificial intelligence techniques, such as artificial neural networks and evolutionary algorithms, is recommended for optimizing design parameters and predicting exposure risk prediction, as explored in Singh et al. [24].

## 5. Conclusions

A quantitative assessment of whole-body vibration exposure was performed using a three-dimensional computational model designed to analyze relevant frequencies, accelerations, and structural deformations. The main conclusions of the study are as follows:

- The computational model allowed a detailed analysis of vibration characteristics across different configurations. Modal analysis revealed that the first eight natural frequencies range between 5 and 13 Hz, a critical band associated with human body resonance.
- The results showed a concerning level of vibration exposure: 86% of the evaluated cases exceeded the alert limit A(8), and in 97% of the situations, the resulting vibration dose value (VDV) also surpassed this limit. Although the A(8) values remained below the maximum allowed by Brazilian regulations, the VDV exceeded this threshold in approximately 20% of cases.
- The developed model shows great potential for future applications in defining control parameters for the design and manufacture of machine cabins and seating systems. It allows early identification of harmful frequency bands, thereby contributing to the development of safer and more ergonomic solutions through computer simulations.

Finally, the proposal recommends several modifications to the seat design, including adjusting the foam density (which affects rigidity and natural frequencies), incorporating shock absorbers into the metal frame, and increasing the seat’s overall mass to change its natural frequency.

## Figures and Tables

**Table 1:** Overview of the most relevant and recent work in WBV with FE model.

Author and Year	Goal	Methodology	Main Results
[36]	WBV assessment and 3D simulation in seated vehicle occupants.	Creation of a 3D human model to predict WBV exposure, and comparison with WBV exposure assessments in the sitting position.	The model allowed the prediction of exposure to WBV up to 30 Hz. The research showed the ability to improve vibration by predicting exposure in the human seat at the start of a new vehicle.
[37]	Develop a biomechanical model with the real anatomy of the human body to understand the human response to vibration.	A finite element model of the seated human body was developed with various segments, and the body mass percentages were compared with previous data. The model was exposed to vibration.	The seated human model served to reflect the transmissibility of the seat on the spine and the main modes of the human body below 10 Hz, which is conducive to expressing the human response to vibration.
[38]	Evaluate the effect of whole-body vibration on various segments of the human body.	A three-layer 3D model was created according to male anthropometric data and exposed to accelerations of 0.5, 1.0, and 1.5 m/s^2^ over a frequency range of 0 to 20 Hz.	It was found that the 4 to 6 Hz frequency range is dominant in both evaluations with maximum effect on the head.
[39]	Develop a tool to assess the risk of musculoskeletal injuries among workers and evaluate its validation.	300 male employees at a steel mill were interviewed.	The results showed that personal and physical parameters are important in predicting injuries. The tool can be used to track and assess the risk of musculoskeletal disorders in people with various personal and occupational properties.
[40]	Evaluate the risk to musculoskeletal health of WBV dumper operators in comparison to the non-exposed group.	WBV measurements were taken on 110 dumper operators in three coal mines. A symptom questionnaire was applied and compared with a group of workers not exposed to WBV.	The prevalence of pain in the lumbar region was 2.52 times higher than in the control group. The Mine-2 case group was 2.0 times as prone to vibration risks compared to Mine-3.

**Table 2:** Criteria for Judging and Decision-Making (NHO09) [42].

A(8) (m/s^2^)	VDV (m/s^1.75^)	Technical Consideration	Recommended Practice
0~0.5	0~9.1	Acceptable	At Least Maintenance of existing condition.
>0.5~<0.9	>9.1~<16.4	Above action level	At least the adoption of preventive measures.
0.9~1.1	16.4~21	Uncertainty region	Adoption of preventive and corrective actions to reduce daily exposure.
Above 1.1	Above 21	Above tolerance limit	Immediate adoption of corrective actions.

**Table 3:** Stiffness, damping, and mass parameters used in the one-dimensional dynamic model.

Number	k_1_ (N/m)	k_2_ (N/m)	b_1_ (Ns/m)	b_2_ (Ns/m)	m_1_ (kg)	m_2_ (kg)	Source
1	62,800		1600		56		[47]
2	70,000		600		25	62.2	[48]
3	24,854	28.723			13	54	[46]
4	32,300		514.11	677.60		60	[45]
5	35,776	38.374	761	458			[49]
6						84	[50]
7					62		[50]

**Table 4:** Mesh parameters [35,41].

	Number of Elements	Type of Elements	Simulation Time (s)	Von-Mises Stress (MPa)
Mesh 1	360,612	SOLID187—3D tetrahedral structural solid with 10 nodes, and SOLID186—3D Structural solid with 20 nodes	353.5	140.23
Mesh 2	414,210	SOLID187—3D tetrahedral structural solid with 10 nodes, and SOLID186—3D Structural solid with 20 nodes	481.5	123.6
Mesh 3	645,031	SOLID187—3D tetrahedral structural solid with 10 nodes, and SOLID186—3D Structural solid with 20 nodes	993.8	123.08

**Table 5:** Comparison between actual acceleration and model-predicted acceleration at the portainer operator’s seat [35].

f (Hz)	Actual Acceleration in the *Z*-Axis (m/s^2^)	Acceleration on the Model in the *Z*-Axis (m/s^2^)	Relative Error (%)
2	0.03	0.02	33.00
3	0.10	0.09	10.00
4	0.11	0.13	18.18

**Table 6:** A(8) and VDV results in Portainer operators.

Portainer Operators	1	2	3	4	5	6	7	8	9	10	11	12
A(8) (m/s^2^)	0.52	0.43	0.52	0.49	0.36	0.51	0.55	0.66	0.57	0.58	0.52	0.51
VDV (m/s^1.75^)	10.64	11.43	12.98	12.92	8.20	12.67	12.54	14.96	12.66	13.01	10.66	12.65

**Table 7:** Stiffness, Damping, and Mass Data Cases.

Nº	k_1_ (N/m)	k_2_ (N/m)	b_1_ (Ns/m)	b_2_ (Ns/m)	m_1_ (kg)	m_2_ (kg)	Fonte
1	62,800	28,723	1600.00	677.60	56	84.0	[38,39]
2	70,000	38,374	600.00	458.00	25	62.2	[40,41]
3	24,854	28,723	514.11	677.60	13	54.0	[37,38]
4	32,300	28,723	514.11	677.60	62	60.0	[37,39]
5	35,776	38,374	761.00	458.00	62	84.0	[41]

**Table 8:** Displacement Analysis.

	Number of Elements	Displacement (mm)	Percentual Difference (%)
Mesh 1	360,612	0.34011	----
Mesh 2	414,210	0.36661	7.79
Mesh 3	645,031	0.36878	0.59

**Table 9:** Modal Participation Factors by Axis.

Mode	Frequency	Modal Participation Factor		
		X	Y	Z
1	5.4048	0.27963	−0.49594 × 10^−2^	−0.24032 × 10^−3^
2	8.1091	−0.27088 × 10^−2^	−0.12503	0.31149
3	9.413	0.78822 × 10^−1^	0.19687	0.16650
4	9.493	−0.10853	0.14911	0.12604
5	9.9703	0.55712 × 10^−2^	0.15575	0.85792 × 10^−1^

**Table 10:** Boundary conditions used in harmonic analysis.

Load	245 kN (Y)
Acceleration	1 m/s^2^ (Z)
Damping (b)	1600 Ns/m
Stiffness (K)	28,723 N/m
Mass (m)	110.5 kg
Damping ratio	0.45

**Table 11:** Harmonic analysis results of the study.

f (Hz)	Displacement (mm)	a (m/s^2^)	f (Hz)	Displacement (mm)	a (m/s^2^)
1	0.16999	0.0097109	6	0.25534	0.3629
2	0.17600	0.027793	7	0.29224	0.56533
3	0.18643	0.089240	8	0.32551	0.82243
4	0.20213	0.127670	9	0.34378	1.0993
5	0.22462	0.221690	10	0.34497	1.3619

## Data Availability

Data are contained within the article.
